# Bioactive Components, Volatile Profile and In Vitro Antioxidative Properties of *Taxus baccata* L. Red Arils

**DOI:** 10.3390/molecules26154474

**Published:** 2021-07-24

**Authors:** Małgorzata Tabaszewska, Agata Antoniewska, Jaroslawa Rutkowska, Łukasz Skoczylas, Jacek Słupski, Radosława Skoczeń-Słupska

**Affiliations:** 1 Department of Plant Product Technology and Nutrition Hygiene, Faculty of Food Technology, University of Agriculture in Cracow, Balicka Str. 122, 30-149 Cracow, Poland; malgorzata.tabaszewska@urk.edu.pl (M.T.); lukasz.skoczylas@urk.edu.pl (Ł.S.); jacek.slupski@urk.edu.pl (J.S.); radoslawa.slupska@urk.edu.pl (R.S.-S.); 2 Institute of Human Nutrition Sciences, Faculty of Human Nutrition, Warsaw University of Life Sciences (WULS-SGGW), Nowoursynowska Str. 159c, 02-776 Warsaw, Poland; jaroslawa_rutkowska@sggw.edu.pl

**Keywords:** *Taxus baccata* L. red arils, ascorbic acid, carotenoid, phenolic compounds, antioxidant potential, volatile compounds

## Abstract

This study aimed at assessing the composition of bioactive compounds, including ascorbic acid, carotenoids and polyphenols, the volatile compound profile and the antioxidant activity of red arils (RAs) of *Taxus baccata* L. grown in diverse locations in Poland. Among the carotenoids assayed in high quantities (3.3–5.42 μg/g), the lycopene content (2.55–4.1 μg/g) was remarkably higher than that in many cultivated fruits. Samples collected from three sites were distinguished by higher amounts of ascorbic acid (125 mg/100 g, on average) than those found in many cultivated berries. Phenylpropanoids quantitatively dominated among the four groups of phenolic compounds. Chromatographic separation enabled the detection of two phenylpropanoid acids: ferulic and *p*-coumaric. Irrespectively of the growth site, RAs contained substantial amounts of (-)-epicatechin (1080 μg/100 g, on average). A higher ability to scavenge DPPH^●^ and ABTS^●+^ radicals was found in the hydrophilic fraction of RAs from two sites (Warsaw and Koszalin) compared with the other two sites. The volatile compound profile of RAs was dominated by alcohols, followed by ketones, esters and aldehydes. The presence of some volatiles was exclusively related to the specific growth site, which may be regarded as a valuable indicator. The combination of bioactive and volatile compounds and the fairly good antioxidant potential of RAs render them an attractive source for preparing functional foods.

## 1. Introduction

A diet rich in berries may protect human beings from oxidant stress. Epidemiological studies indicated that consumption of berries was linked with a decreased risk of cardiovascular diseases, diabetes and certain types of cancer [1]. Many studies revealed that wild berries and other unique fruits derived from natural habitats were rich in secondary metabolites [2,3,4,5,6]. For example, *Plinia trunciflora*, *Vaccinium myrtillus, Rubus chamaemorus, Hippophaë rhamnoides* (sea buckthorn) and *Amelanchier alnifolia* (Saskatoon berries) contain amazingly high amounts of phenolic compounds (anthocyanins, flavonoids, phenolic acids, flavonols or tannins) [2,4,5,6,7]. Wild berries may also be regarded as a substantial source of vitamin C, carotenoids and other valuable compounds [5,7]. These compounds represent not only proved antioxidant properties in vitro but are also involved in upregulation of the genes coding for the antioxidant enzymes MnSOD and GPx [6].

Except for genotype attributes, the composition of berries is substantially dependent on microclimate and local environmental factors, e.g., soil fertility, temperature, light conditions, volume and frequency of precipitation. Bilberries collected at sunny sites with no topsoil damage contained more phenolic compounds than samples from dense forests or sites with visible soil erosion [4,8]. Ma et al. [2] reported that the content and profile of flavonol glycosides were strongly affected by the altitude and/or latitude of growth sites. Jaakkola et al. [5] suggested that the amount of sunlight, temperature levels and rainfall could be the main factors affecting the chemical composition, especially anthocyanin, of cloudberries. Additionally, in our previous study, we noted the contents of macronutrients, fatty acids, amino acids and macro- and micro-elements in the seedless red fleshy part of the berries of *Taxus baccata*, known as red arils (RAs), to be site-related and affected by water availability (sum of precipitation), sunlight intensity and soil parameters and composition [9].

For example, the protein content in RAs was higher than in cultivated berries [10]. RAs can also be regarded as a novel dietary source of valuable PUFAs belonging to the n-3 family, and the unique polymethylene-interrupted fatty acids, such as pinolenic, sciadonic and juniperonic acids. 

Except for RAs, the morphological parts of the *Taxus baccata* plant (twigs, leaves, root barks, stems) contain diterpene alkaloids known as taxines. Some of these compounds (taxines A and B) are responsible for its cardiotoxicity, whereas others (paclitaxel) are highly appreciated as important naturally occurring anticarcinogens [11,12]. Additionally, a recently published study confirmed that RAs were free of toxic compounds [9]. 

A wide range of phenolic compounds were identified in different parts of *Taxus* species plants. For example, needles of the Himalayan yew *Taxus baccata* were found to contain several phenolic compounds, including 3-demethyl-(-)-secoisolariciresinol, a lignan, and taxuside, a phenolic glucoside [13]. In *Taxus* species (*T. chinensis*, *T. cuspidata* and *T. media*), the twigs and leaves are of high interest due to the presence of health-beneficial flavonoids, with a substantial share of isoquercitrin, quercitrin, bilobetin and sciadopitysin [12]. Fourteen flavonoids were identified on the surface of needles of *Taxus baccata*. Among them, 3-O-rutinoside myricetin, 3-O-rutinoside quercetin and quercetin dominated [14]. Five lignans, which are dimeric phenylpropanoids, were identified in the heartwood of *Taxus baccata*: lariciresinol, 3’-demethylisolariciresinol-9’-hydroxyisopropylether, taxiresinol and 3-demethylisolariciresinol [15].

The beneficial properties of phenolic compounds derived from different parts of the *Taxus baccata* plant were reported by many authors. For example, Milutinović et al. [16] reported that the leaves and seed cones of *Taxus baccata* were a potential source of phenolic compounds, especially flavonoids, having antioxidant, cytotoxic and strong proapoptotic properties. The methanolic extract of leaves produced a higher cytotoxic effect than that of seed cones [16]. Moreover, lignans isolated from *Taxus baccata* heartwood exhibited significant anti-inflammatory and antinociceptive activities [17]. The ethanol extract of *Taxus baccata* heartwood (2 mg/mL) proved highly active against some Gram-negative bacteria (*Salmonella typhi*, *Pseudomonas pseudomalli*, *Enterobacter cloacca*), as compared with ampicillin and tobramycin. Lignans obtained from *Taxus baccata* also showed a moderate inhibitory activity against butyrylcholinesterase and lipoxygenase, which play a role in the pathogenesis of Alzheimer’s disease [17]. 

Despite the well-studied composition and properties of phenolic compounds of the leaves, bark, heartwood and cones of the *Taxus baccata* plant, the profile of phenolic compounds of RAs remains to be studied. To our knowledge, no study on the volatile components in RAs has been reported. Thus, the main goal of this study was to assess the profile of bioactive compounds (polyphenols, carotenoids, ascorbic acid) and the antioxidant potential of RAs collected at different sites in Poland, as potential functional food ingredients. Complementarily, volatile profiles of RAs were studied to identify the main components responsible for their pleasant and characteristic fruit flavor.

## 2. Results and Discussion

### 2.1. Bioactive Compounds 

Red arils proved to be rich in carotenoids (3.30–5.42 μg/g; Table 1) and markedly differed in the lycopene content (2.55–4.1 μg/g) depending on the growth site (*p* ˂ 0.05). The latter was remarkably higher than in peach, apricot, Ruby Red grapefruit, pumpkin and muskmelon; only tomatoes and rose hip fruits exceeded RAs in that respect [18,19]. The substantial share of lycopene probably had a great impact on the red color of RAs.

**Figure 1:**
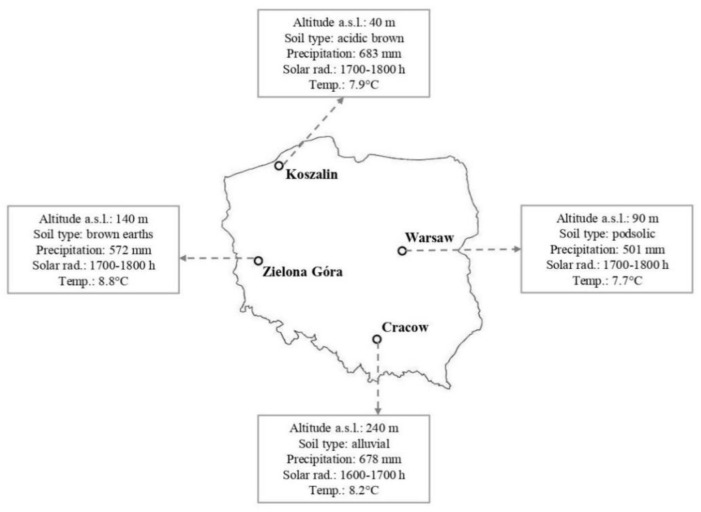
Location and main environmental parameters of collection sites of *Taxus baccata* red berries in Poland. Abbreviations: Altitude a.s.l.—altitude above sea level; Precipitation—total annual precipitation; Solar rad.—exposure to solar radiation; Temp.—average annual temperature.

The content of ascorbic acid (109.5–145 mg/100 g) in samples collected from three sites was remarkably higher than in many other berries: cloudberries (56–80 mg/100 g), strawberries (90.1 mg/100 g), chokeberries (13.2 mg/100 g) and blueberries (12.8 mg/100 g), and in other fruits: orange (76 mg/100 g), lemon (55.5 mg/100 g), pineapple (70.3 mg/100 g) and *Rosa dumalis* hips (65.75 mg/100 g) [3,5,10,20]. It should be noted that RAs derived from four growth sites differed in the content of ascorbic acid (Table 1, Figure 1), probably due to the diverse environmental conditions, such as temperature, water availability, sunlight and wind exposure [21].

The spectrophotometric assay enabled detecting phenolic compounds from three categories: phenylpropanoids, flavonols and anthocyanins, in RAs (Table 2). It is worth pointing out the considerable amounts of phenylpropanoids, compounds having a C6-C3 carbon skeleton as the core structure and phenolic acids, being one of the main categories there. These compounds are known to have multifaceted effects which include antimicrobial, antioxidant, anti-inflammatory, antidiabetic and anticancer activities, and to exhibit renoprotective, neuroprotective, cardioprotective and hepatoprotective effects [22]. The content of phenylpropanoids was significantly (*p* ˂ 0.05) dependent on the collection site, the highest content being found in RAs from the northern site (Koszalin): 65.3 mg CA/100 g, and the lowest from the western site (Zielona Góra): 39.2 mg CA/100 g. The substantial presence of phenylpropanoids in the ethanolic extract of the heartwood of *Taxus baccata* was also reported by Erdemoglu et al. [15].

Chromatographic separation enabled detecting two phenolic acids from the phenylpropanoid category: ferulic and *p*-coumaric acids. Samples collected from the western site (Zielona Góra) showed the lowest content of these compounds compared with the other three sites (Table 2). These compounds have attracted considerable attention because of their broad biological and pharmacological effects, due to their antioxidant effects. Ferulic, *p*-coumaric and caffeic acids exhibited an immunomodulatory effect which could be ascribed, in part, to their cytoprotective effect via their antioxidant capacity [23]. Having in mind the beneficial role of phenolic acids for human health, RAs contained from 140 to 170 µg/100 g of gallic and protocatechuic acids combined (except samples from the Zielona Góra site). It should be noted that phenolic acids were not previously detected in various parts of the *Taxus* genus [12,14,17,24].

The presence of flavonol compounds (e.g., quercetin; Q) in *Taxus* species twigs and leaves was reported by others [12,14]. Epidemiological studies indicated that flavonol intake was associated with a reduced risk of cancer, coronary heart disease and stroke [25]. Red arils from the northern site had higher amounts of flavonols than samples from the other sites (53.6 mg Q/100 g and 36.1 mg Q/100 g, respectively) and rose hips [3].

Chromatographic separation enabled identifying (-)-epicatechin, an important flavonoid compound. It has been demonstrated that catechin and epicatechin reduced mitochondrial dysfunction and oxidant stress induced by amiodarone in human lung fibroblasts [26]. No significant between-region differences were found in the (-)-epicatechin content, which averaged 1080 µg/100 g. It was much higher than in strawberry, peach and plum, but much lower than in apple, pear, raspberry, blackberry and black grape [27].

The spectrophotometric assay enabled determining total flavonoids in RA samples. The literature data confirm a substantial share of flavonoids occurring in the needles of the *Taxus* genus [14]. Flavonoids exhibit cardiovascular protection despite being poorly absorbed; they are metabolized by the intestinal microbiota into various phenolic acids [28]. RAs from the Warsaw site differed from the other three sites in the content of flavonoids (21.1 and 10.5 mg C/100 g, respectively) and of anthocyanins (59.3 and 35.9 mg Cy/100 g, respectively). These values are similar to those reported by Drkenda et al. [29] for cornelian cherry fruits, and for blackberries by de Souza et al. [10]. RAs proved to be a much better source of anthocyanins than other berries: red raspberries, blueberries and cherries [10]. Our findings are in accordance with reports stating that the levels of phenolics in fruits, namely, anthocyanins, are environmentally dependent [4]. In particular, flavonoids and anthocyanins are upregulated by exposure to harsh weather conditions or increased UV irradiation [4]. Additionally, as pointed out by Drkenda et al. [29], regional differences in the phenolic content may have been due to differences in day/night temperatures, which can affect the anthocyanin accumulation in some fruits. As in the case of lycopene, the substantial abundance of anthocyanins contributed to the red color of RAs. 

The total phenolic content ranged from 25.7 to 53.8 mg GAE/100 g. The samples from the Warsaw and Koszalin sites, which contained higher amounts of phenylpropanoids and anthocyanins, also showed a higher total phenolic content than samples from the other two sites (Table 2). A similar total phenolic content was reported for many cultivated fruits: apple (red delicious), banana, grape (green and red), nectarine and pear [30]. However, as compared with the literature data, the total content of phenolic compounds in RAs was not higher compared with other cultivated and wild berries (blackberries, red raspberries, blueberries, bilberries, *Rubus chamaemorus* berries) [5,8,10].

### 2.2. Antioxidant Potential

It is well known that non-polar compounds such as carotenoids and polar ones such as ascorbic acid and phenolics contribute to the antioxidant activity of fruits. Our results reveal that RAs contained substantial amounts of all those compounds (Table 1 and Table 2). Assays of the radical scavenging activity (DPPH^●^ and ABTS^●+^) were conducted in order to provide new information on the antioxidant activity of both fractions of RAs. As shown in Table 1, the hydrophilic fraction revealed a much higher free radical scavenging ability assayed by DPPH^●^ and ABTS^●+^ than the lipophilic one. The antioxidant activity of the hydrophilic fraction of RAs, as determined by DPPH^●^, ranged from 6.18 to 11.26 μM TE/g. Higher values of DPPH^●^ were found in samples from Warsaw and Koszalin, containing higher amounts of total polyphenols and other phenolic compounds such as phenylopropanoids, compared to the other two sites (Table 2), and the same was true for the antioxidant potential assayed by ABTS^●+^. These observations are in agreement with other reports stating that phenolics are the primary compounds which determine the antioxidant potential of plants [30,31]. However, our results are at variance with other reports stating that the contents of most antioxidant phenolic compounds are highly correlated with sun exposure and location altitude [5]; the contents of all phenolic compounds and the antioxidant potential of RAs from the Cracow site (highest altitude among the studied sites) were lower than in samples from the northern site (Koszalin). Stressful environmental conditions, such as low/high temperature extremes, heavy metals and drought, probably bring about an increase in the level of flavonoids, which may reduce the negative effects of free radicals generated by diverse environmental stressors [9,32]. On the other hand, Cocco et al. [21] found the highest total antioxidant capacity in strawberry fruits from a northern location characterized by a lower temperature before harvest, which agrees with our observations. The higher antioxidant potential of the hydrophilic extract of RAs from Warsaw and Koszalin than from the other two sites was also confirmed by FRAP assays (Table 1).

The antioxidant capacity of the hydrophilic fraction of RAs was lower than other berries, probably because of the lower presence of total polyphenols and anthocyanins, and the absence of other valuable phenolic compounds occurring in large amounts in blue-colored berries (blackberry, blueberry, chokeberry) [10,33,34]. However, our results of the antioxidant activity of the hydrophilic RA fraction are promising as compared with the antioxidant potential of many cultivated fruits regarded as valuable sources of antioxidant compounds in the human diet (apple, citrus, grapes, pear, banana, cantaloupe) [30].

As shown in Table 1, the significantly (*p* ˂ 0.05) differentiated antioxidant potential of the lipophilic RA fraction was related to the carotenoid content. The samples from the Zielona Góra site, which contained low amounts of carotenoids, also had a low ability to scavenge ABTS^●+^ and DPPH^●^ radicals, as compared with the other three sites. It should be noted that, irrespectively of the growth location, the antioxidant capacity of the lipophilic RA fraction was comparable or higher than that found in tomato varieties, regarded as the crucial source of lycopene in the human diet [35].

### 2.3. Volatile Compound (VC) Profile

The GC/MS analysis revealed a complex mixture of 63 VCs in the headspace of RAs (Table 3). Their abundance and content significantly (*p* ˂ 0.05) depended on the collection site. RA samples from Koszalin contained more VCs compared to the other three sites (56 and 44–45). To our best knowledge, the VC profile of RAs has not been studied thus far, in contrast to studies in which the VCs of the needles (leaves) or twigs of *Taxus baccata* L. and *Taxus canadensis* were analyzed [36,37].

Alcohols quantitatively dominated in the VC profile of RAs, with their highest content (48.77%) and the greatest diversity found in the samples from the Koszalin site. The leaves of *Taxus baccata* L., originated from different sites in Serbia, dominated the share of alcohols (30.8–50.1%) [37]. Among seventeen alcohols, five dominated quantitatively: ethanol, 1-hexanol, 3-methyl-1-butanol, 2,3-butanediol and benzenemethanol. Alcohols, as well as aldehydes, may be derived from unsaturated fatty acids in fruit tissues via oxygenation and sequential transformation of hydroperoxides, catalyzed by lipoxygenase [38]. During ripening, cell walls and membranes may become more permeable, letting the lipoxygenase pathway become active without tissue disruption [39].

Alcohols contribute to the fruity odour [39]. For example, 1-hexanol has a pleasant floral fragrance and is found in the plum, cherry and blueberry; 1-octanol has a fruity fragrance which occurs frequently in the cherry, peach and nectarine. The fruity odour in the elderberry is related to 3-methylbutan-1-ol and 1-pentanol [40,41]. 1-Butanol, 2-methyl also occurs in, e.g., apples, grapes, tomatoes and elderberry varieties [41]. Other alcohols such as 1-hexanol, 1-octanol and 3-hexen-1-ol are considered to be important contributors to the green grass aroma of fruits [41].

The total alcohol content was significantly (*p* ˂ 0.05) dependent on the growth site (Table 3). RAs from the Zielona Góra and Koszalin sites had higher relative total contents of alcohols than those of the other two sites (Cracow and Warsaw). This is mainly attributed to the higher abundance of 2,3-butanediol and benzeneethanol, and 1-hexanol and bezenemethanol, in samples from the Zielona Góra and Koszalin sites, respectively. As with RAs from the Koszalin site, benzenemethanol content (5.17%) was also detected in different varieties of cherries [42]. The substantial presence of alcohols in fruits may be an indicator of the ending maturation of fruits [39].

Ketones were the second important chemical group in RAs, their total content ranging from 10.83 to 21.02%, depending on the growing location. A dominating share of 1-propanone was found in *Taxus Canadensis* leaves [36]. Among seven ketones, four were most abundant: 2,3-butanedione, 3-hydroxy-2-butanone (acetoin), 4-ketoisophorone (2,2,6-trimethyl-2-cyclohexene-1,4-dione) and 3-octanone. Such a rich abundance of ketones in the VC profile of RAs is unusual in common fruits such as apples, pears, bananas, strawberries and pineapples [39], while high amounts of ketones, especially 4-ketoisophorone, are present in the VC profile of saffron, the dried red stigmata of *Crocus sativus* L. flowers [43,44]. The structure of 4-ketoisophorone is similar to that of safranal (2,6,6-trimethyl-1,3-cyclohexadiene-1-carboxaldehyde), which makes it responsible for the saffron aroma [44]. Isophorone biosynthesis is associated with the degradation of zeaxanthin [43].

Depending on the growth site, RAs differed greatly (*p* ˂ 0.05) in the contents of two ketones providing a buttery, creamy aroma: 3-hydroxy-2-butanone (acetoin; 1.72–9.75%) and 2,3-butanedione (1.52–5.17%). According to the literature data, differences in the content of 3-hydroxy-2-butanone in RAs could be due to differences in the stage of maturity of samples from different sites [45,46]. The increased content of 3-hydroxy-2-butanone in acerola and mangaba fruits was attributed to the late stage of maturation [45,46].

Aldehydes in RAs were represented by seven VCs, their total amounts being lower (4.70–7.34%) than those of ketones; the dominating aldehydes were hexanal and benzaldehyde, independently of the growth site. Two C6 aldehydes: hexanal and 2-hexenal, are synthesized via the lipoxygenase pathway from C18 PUFAs: linoleic and α-linolenic [39,47]. Nonanal, decanal and benzaldehyde occurred in RAs from all sites. They are potentially important odorants in the Chinese dwarf cherry, plum and grape [40]. The occurrence of benzaldehyde (1.10–3.42%) was potentially attributed to the candy sweet note of rose hips and elderberry fruits [3,41]. 

As many as 13 esters were found in RAs, their relative total content ranging from 2.08 to 8.84%, depending on the growth site (Table 3). Much more esters were found in the Chinese dwarf cherry (35 VCs) and Chinese quince (*Pseudocydonia sinensis;* 66 VCs) than in RAs [38,40]. Esters are well-known as major contributors to the characteristic fruity and sweet aromas of a wide variety of fruits [39]. Four esters (methyl acetate, ethyl acetate, methyl hexanoate and ethyl hexanoate) were detected in RAs from all studied sites, and those from the Zielona Góra site had a much higher relative content of ethyl acetate (up to 5.58%) than samples from the other sites; this might have been due to a late stage of maturation of RAs, as in the case of *Arbutus unedo* L. fruits, as supported by the presence of ethyl hexaonate in RAs [47]. Hexyl acetate, however, abundant only in minor amounts in samples from two sites, may provide a sweet, fruity fragrance that is rich in apricots, peaches, apples and Chinese draft cherries [40].

Among the terpenoid compounds, eight monoterpenes and four terpenoids were identified in RAs (Table 3). Depending on the growth site, they accounted for 2.07–6% of the total VCs and gave the pleasant floral and fruit aromas of RAs [40]. Greater diversity in terpenoids was found in the rose hip species (27 VCs) than in RAs [3]. Five of twelve terpenoids (D-limonene, α-pinene, β-pinene, p-cymene) were found in RAs from all sites. These terpenes were also detected in *Taxus baccata* L needles [37]. Among them, the content of D-limonene, which gives the fruity and citrus flavor, dominated (1.33–2.87%).

Some monoterpenes are nutraceuticals and have other beneficial functions. For example, α-pinene and limonene revealed bioactive properties such as antioxidant, antimicrobial or antiulcer activities [3]. Rozza et al. [48] showed that α-pinene had an antispasmodic effect on the rat’s ileum and could induce antinociceptive actions. Limonene also showed an interesting chemo-preventive activity against gastric colorectal and other types of cancer [49]. The antimicrobial activity of essential oils rich in limonene, p-cymene, α-pinene and β-pinene against a wide range of bacteria is worth mentioning [50].

Unlike monoterpenes, terpenoids were rarely present in the RA samples; myrtenol, a marker VC in *Taxus baccata* L needles, was found in RAs from three locations (Table 3) [37]. Other terpenoids such as 4-thujanol, terpinen-4-ol and menthol were detected in samples from the Koszalin and Cracow sites (Table 3). Some beneficial properties of RA terpenoids have been reported. Myrtenol was shown to be an antioxidant in vitro: it prevented lipid peroxidation and removal of the hydroxyl radical and of nitrite ions [51]. Badary [52] investigated the cytotoxicity of terpinen-4-ol against two different colon (DLD-) and lung (A-549) cell lines.

Other compounds of minor occurrence in the volatile profile of RAs were hydrocarbons and lactones. Among four hydrocarbons, only dodecane was found in RAs from all sites. The trace amounts of butyrolactone probably had no effect on the flavor of RAs.

Summing up, the main VCs present in RAs were alcohols, followed by ketones, esters and aldehydes. Thirteen VCs were most abundant in samples of all sites: ethanol; 1-hexanol; benzenemethanol; 1-butanol-3-methyl; 1-butanol-2-methyl; 2-butanone-3-hydroxy; 2,3-butanedione; 2,6,6-trimethyl-2-cyclohexene-1,4-dione; ethyl acetate; hexanal; benzaldehyde; and D-limonene. Some VCs were specific to the growth sites of RAs: menthol-Cracow site; 3-pentanone, 4-thujanol, 4-carvomenthenol and styrene - Koszalin site; ethyl isobutanoate-Zielona Góra site. Moreover, high amounts of 2,3-butanediol and 1-hexanol proved to be specific to the Zielona Góra and Koszalin sites, respectively.

## 3. Material and Methods

### 3.1. Sampling

Red berries of *Taxus baccata* were collected from plants growing in natural habitats at four localities in Poland: Zielona Góra (15°30′ E, 51°56′ N), Warsaw (21°01′ E, 52°13′ N), Koszalin (16°11′ E, 54°11′ N) and Cracow (19°58′ E, 50°05′ N). In each site, red berries were harvested thrice from ten trees each (from different parts of the crown) growing in three places (*n* = 9). The environmental conditions of the fruit collection are presented in Figure 1. Specific descriptions and characteristics of growing locations are described elsewhere [9]. Fruits were manually separated from the seeds to obtain RAs for analyses.

### 3.2. Red Arils Extract Preparation

#### 3.2.1. Extraction of Hydrophilic Fraction

Ethanolic extracts of RAs were prepared by mixing 10 g of sample with 100 mL of ethanol/water (80:20, *v*/*v*) and homogenized using a DI 25 homogenizer (Ika-Werke, Staufen, Germany). The homogenates were centrifuged at 15,000 rpm for 20 min (MPW-260R centrifuge, MPW Med. Instruments, Warsaw, Poland), and the supernatants were collected for evaluation of antioxidant capacity and main groups of phenolic compounds by applying spectrophotometric assays.

#### 3.2.2. Extraction of Lipophilic Fraction

For the lipophilic ABTS^●+^ and DPPH^●^ antioxidant assays, 3 g of red arils was vigorously shaken with 200 mL of acetone/hexane mixture (4:6) and then thoroughly homogenized (DI 25 homogenizer, Ika-Werke, Staufen, Germany). The homogenates were filtered through Whatman No. 4 filter paper, and the supernatants were used for analysis.

### 3.3. Spectrophotometric Assays of Phenolic Compounds

#### 3.3.1. Total Phenolics Content (TPC)

The TPC was determined spectrophotometrically at 725 nm (Hitachi U-2900 UV-Vis spectrophotometer, Hitachi, Tokyo, Japan) by reduction of phosphotungstic-phosphomolybdic acid (Folin-Ciocalteu’s reagent) to blue pigments in alkaline solution according to a modified method of Singleton and Rossi [53]. Briefly, 0.1 mL of RA ethanolic extract was diluted with 7.9 mL of deionized water, and 0.5 mL of Folin-Ciocalteu’s reagent and 1.5 mL of 20% sodium carbonate solution were added and mixed thoroughly. The mixture was kept in a water bath at 40 °C for 30 min. The results were expressed as gallic acid equivalents (GAE; mg GAE/100 g of RAs fresh weight). 

#### 3.3.2. Total Flavonoids Content

The total flavonoids content was measured by the aluminum chloride colorimetric assay at 510 nm (Hitachi U-2900 UV-Vis spectrophotometer, Hitachi, Tokyo, Japan) [54,55]. An aliquot of 1 mL of RA ethanolic extract was diluted with 4 mL of pure water, and 0.3 mL of 5% NaNO_2_ and 0.3 mL of 10% AlCl_3_ were added. Then, after 6 min, 2 mL of NaOH (1M) and water were added up to 10 mL of the total sample volume and mixed thoroughly. The total flavonoids content was expressed as mg catechin (C)/100 g of RAs fresh weight.

#### 3.3.3. Total Phenylpropanoids, Total Flavonols and Total Anthocyanins Content

The contents of the main groups of phenolic compounds such as phenylpropanoids, flavonols and anthocyanins were determined by measuring UV/Vis absorbance according to Fukumoto and Mazza [25]. Reaction mixture: 0.25 mL of RA ethanolic extract with 0.25 mL of 0.1% HCl in 96% ethanol and 4.5 mL of 2% HCl. The absorbance of the solution was read at 320 nm, 360 nm and 520 nm to measure phenylpropanoids, flavonols and anthocyanins, respectively. The results were converted using the molar absorbance (ɛ) of the respective standards: caffeic acid (CA; 0.887 M^−1^cm^−1^) for phenylpropanoids, quercetin (Q; 0.513 M^−1^ cm^−1^) for flavonols and cyanidin (Cy; 0.645 M^−1^cm^−1^) for anthocyanins, and expressed as mg CA, Q or Cy per 100 g of RAs fresh weight. 

#### 3.3.4. Total Carotenoids Content

The total content of carotenoids (TCC) was determined spectrophotometrically (Hitachi U-2900 UV-Vis spectrophotometer, Hitachi, Tokyo, Japan) according to the PN-EN 12136 method [56]. To precipitate carotenoids, 1.5 g of the homogenized sample was treated with Carrez I and II solutions, mixed and then centrifuged. Extraction of carotenoids was carried out three times with 25 mL of acetone; petroleum ether (45 mL) was added to the supernatant and mixed thoroughly. The absorbance of ether extract was measured at 450 nm using petroleum ether as a blank sample. The TCC (mg/100 g of fresh weight) was calculated according to the following equation: TCC = A × V × 10^6^/A^%^_1 cm_ × 1000 × m, where: A—absorbance of the ether extract at 450 nm; V—volume of extract (ml); A^%^_1 cm_—extinction coefficient of carotenoids in petroleum ether solution; m—sample weight (g).

#### 3.3.5. Lycopene and β-carotene

Lycopene and β-carotene from RAs were extracted by using a mixture of acetone–hexane (4:6) according to the method of Nagata and Yamashita [57]. The absorbance of the filtrate was measured spectrophotometrically at 453, 505, 645 and 663 nm (Hitachi U-2900 UV-Vis spectrophotometer, Hitachi, Tokyo, Japan). The contents of lycopene and β-carotene were computed from the following equations: lycopene (mg/100 mL of extract) = −0.0458 × A_663_ + 0.204 × A_645_ + 0.372 × A_505_ − 0.0806 × A_453_; β-carotene (mg/100 mL) = 0.216 × A_663_ − 1.220 × A_645_ − 0.304 × A_505_ + 0.452 × A_453_. The results were expressed as mg/100 g of fresh weight. 

### 3.4. Chromatographic Analysis of Bioactive Compounds

#### 3.4.1. Ascorbic Acid

Samples were prepared according to the PN-EN 14130 instructions [58]. Chromatographic analysis was carried out using the HPLC Dionex UltiMate 3000 system (Thermo Scientific, Germering, Germany) equipped with the Velocity C18 PLMX 250 × 4.6 mm, 5 µm column (Bionacom LTD, London, Great Britain) and the Velocity C18 PLMX, 3.0–4.6 mm, 5 µm precolumn of the same company. The mobile phase was a mixture of aqueous solution of 0.1% *meta*-phosphoric acid (*v*/*v*) and was used at an isocratic flow rate of 1 mL/min. Ascorbic acid was detected using a DAD detector (Thermo Scientific, Germering, Germany) at 254 nm wavelength. The analysis lasted 20 min, and the signal was recorded at 254 nm. The content of ascorbic acid was calculated on the basis of a standard curve and expressed as mg/100 g of fresh weight.

#### 3.4.2. Phenolic Profile

In order to determine free and bound phenolic compounds before extraction, RA samples were hydrolyzed with 2M NaOH, mixed with a Labnet vortex mixer (Labnet International, Inc., Edison, NJ, USA) and left in a darkroom at ambient temperature (20–22 °C) for 4 h. Afterwards, the samples were neutralized to pH 2.1–2.6 with 2M HCl according to Klimczak et al. [59]. Phenolic compounds were extracted using methanol (HPLC-gradient) solution with 1% L-ascorbic acid. Prior to the analysis, the samples were centrifuged and filtered. Other details about the preparation of samples are presented elsewhere [60].

The chromatographic analysis was conducted using an HPLC Dionex UltiMate 3000 system with a DAD detector (Thermo Scientific, Germering, Niemcy) and a Cosmosil 5C18-MS-II, 250 × 4.6 mm ID, 5 µm column (Nacalai Tesque, INC, Kyoto, Japan). The mobile phase consisted of two eluents: A—2% (*v*/*v*) aqueous solution of acetic acid, and B—100% methanol, and was used at a flow rate of 1 mL·min^−1^. The chromatographic analysis was carried out for 50 min in the following eluent system: eluent A—0 min 95%; 10 min 70%; 25 min 50%; 35 min 30%; 40 min 95%; 50 min 95%, until the end of analysis. Calibration curves were prepared for the following standards: protocatechuic acid, (+), (-)-epicatechin, ferulic acid, *p*-coumaric acid (Sigma Aldrich, Taufkirchen, Germany), gallic acid (Merck, Darmstadt, Germany). The results were expressed as µg/100 g of RA fresh weight.

### 3.5. Determination of Antioxidant Activity 

The antioxidant activities of the hydrophilic and lipophilic fractions of RAs were expressed as Trolox equivalent antioxidant capacity (TE μM) per 1 g of fresh weight of samples.

#### 3.5.1. DPPH^●^ Radical Scavenging Activity

The free radical scavenging capacity of RA extracts on 1,1-diphenyl-2-picrylhydrazyl radicals (DPPH^●^) was determined using the modified method of Sánchez-Moreno et al. [61]. Briefly, 1 mL of DPPH^●^ solution, 2.9 mL of methanol and 0.1 mL of red aril extract were vigorously mixed and incubated in the dark for 30 min at ambient temperature. Then, the absorbance was measured at 515 nm against a blank (mixture without extract) using a UV/Vis spectrophotometer (Specord 40, Analytik Jena AG, Jena, Germany).

#### 3.5.2. ABTS^●+^ Radical Cation Scavenging Activity

The ABTS 2,2-azinobis-(3-ethylbenzothiazoline-6-sulfonic acid) radical cation scavenging activity of RA extracts was measured spectrophotometrically using a modified method of Re et al. [62] and Serpen et al. [63]. Briefly, to obtain ABTS^●+^, 5 mL of ABTS stock solution (7 mM) and 5 mL of potassium persulfate solution (2.45 mM) were kept in the dark at ambient temperature for 16 h before use. The ABTS^●+^ solution was diluted with ethanol until an absorbance of 0.7 was obtained (A_734_ = 0.700 ± 0.02). An aliquot of 750 μL of RA extract was added to 3 mL of ABTS^●+^ solution and mixed, and the absorbance at 734 nm was measured 6 min later (against blank) using a UV/Vis spectrophotometer (Specord 40, Analytik Jena AG, Jena, Germany). 

#### 3.5.3. The Ferric-Reducing Antioxidant Power (FRAP)

The FRAP assay was based on measuring the iron-reducing capacities of the samples and was carried out according to the Benzie and Strain [64]. Briefly, the FRAP reagent consisted of 20 mM/L iron (III) chloride solution, 10 mM/L TPTZ (2,4,6-Tri(2-pyridyl)-S-triazine) solution in 40 mM/L HCl and 300 mM/L of sodium acetate buffer (pH 3.6), at a volume ratio of 1:1:10, respectively. FRAP reagent was prepared daily and warmed by incubating in a water bath at 37 °C for 10 min before use. Then, 0.1 mL of the extract was mixed with 3 mL of FRAP solution, the absorbance being measured at 593 nm against a blank, using a Hitachi U2900 UV-Vis spectrophotometer (Hitachi, Tokyo, Japan).

### 3.6. Analysis of Volatile Compounds

The extraction of volatile compounds (VCs) from RA samples was performed by solid-phase microextraction (SPME) using a fiber coated with 50/30 μm DVB/CAR/PDMS divinylbenzene/carboxen/polydimethylsiloxane (Supelco Bellefonte, PA, USA). The VCs were extracted from the headspace of a 5 g sample at 40 °C for 40 min using a conditioned SPME fiber. The 6890N gas chromatograph equipped with a 5795 N mass-selective detector (GC/MS) (Agilent, Santa Clara, USA) was used. The VCs were separated on an HP-5MS column (30 m × 0.25 mm × 0.25 μm film thickness (5%-diphenyl-95%-dimethylpolysiloxane; Agilent, Santa Clara, USA). Helium was used as the carrier gas with a flow rate of 0.9 mL/min. The oven temperature was held at 38 °C for 10 min, then increased up to 200 °C at 4 °C/min and held for 2 min, finally being raised to 250 °C, at 20 °C/min. MS was operated in the electron ionization mode at 70 eV with a scan range of *m*/*z* 33-350 and multiplier voltage of 1670 V. Volatile compounds were identified by comparing their mass spectra with those of the NIST.08 and Wiley 8-th Ed (National Institute of Standards and Technology, Gaithersburg, MD, USA) libraries and by computing the retention index relative to a series of standard alkanes (C6-C20, Kovats indexes). Quantities of VCs were reported as a relative percentage of the total peak area.

### 3.7. Statistical Analysis

The results were presented as means and standard deviations (SD) and were subjected to one-way ANOVA followed by Tukey’s test. Differences between mean values were considered significant at *p* < 0.05. The Statistica 3.1 software (Statsoft, Inc., Tulusa, OK, USA) was used. 

## 4. Conclusions

The bioactive volatile compound profile and *in vitro* antioxidative properties of *Taxus baccata* L. red arils were evaluated for the first time. This study demonstrates that red arils are ascorbic acid-rich berries. Thus, red arils may be regarded as a novel source of vitamin C, fully providing the recommended dietary allowance for healthy adults. Among the carotenoids assayed in high quantities, the lycopene content was remarkably higher than that in many cultivated fruits. Red arils proved to be a fairly good source of phenolic compounds as compared with other berries. It should be pointed out that the antioxidant potential of the hydrophilic fraction revealed a much higher radical scavenging ability (DPPH^●^ and ABTS^●+^ assays) than the lipophilic fraction. The volatile compound profile of red arils was dominated by alcohols, followed by ketones, esters and aldehydes. However, the presence of some volatiles was related exclusively to the specific growth site, which may be regarded as a valuable indicator.

Our findings suggest that the site differentiation in the contents of carotenoids, ascorbic acid, polyphenols and volatile compounds and in the antioxidative activity of red arils resulted from diversified environmental conditions (temperature extremes, water availability, sunlight intensity, soil parameters).

## Figures and Tables

**Table 1:** Bioactive components and antioxidative potential of red arils (mean ± SD).

Component	Fruit Collection Site			
	Zielona Góra	Warsaw	Koszalin	Cracow
Component (mg/100 g of fresh weight)				
Ascorbic acid	109.5 ± 4.2 ^b^	145.0 ± 4.9 ^d^	119.8 ± 2.2 ^c^	60.7 ± 1.4 ^a^
β-carotene	0.15 ± 0.05 ^a^	0.20 ± 0.03 ^a^	0.13 ± 0.02 ^a^	0.44 ± 0.06 ^b^
Lycopene	2.57 ± 0.07 ^a^	3.11 ± 0.32 ^a,b^	4.10 ± 0.65 ^b^	2.55 ± 0.09 ^a^
Carotenoids	3.30 ± 0.09 ^a^	4.28 ± 0.22 ^b^	5.42 ± 0.54 ^c^	4.88 ± 0.32 ^b^
Antioxidant activity of hydrophilic fraction (µM TE/g of fresh weight)				
ABTS^•+^ assay	2.45 ± 0.14 ^a^	3.84 ± 0.22 ^c^	4.21 ± 0.33 ^c^	3.13 ± 0.11 ^b^
DPPH^•^ assay	6.18 ± 0.29 ^a^	8.53 ± 0.57 ^b^	11.26 ± 1.24 ^c^	6.70 ± 0.39 ^a^
FRAP assay	1.37 ± 0.06 ^a^	2.62 ± 0.06 ^c^	2.67 ± 0.05 ^c^	1.78 ± 0.04 ^b^
Antioxidant activity of lipophilic fraction (µM TE/g of fresh weight)				
ABTS^•+^ assay	0.26 ± 0.06 ^a^	0.68 ± 0.15 ^c^	0.74 ± 0.18 ^c^	0.54 ± 0.10 ^b^
DPPH^•^ assay	0.22 ± 0.05 ^a^	0.42 ± 0.10 ^c^	0.51 ± 0.14 ^d^	0.36 ± 0.11 ^b^

TE—Trolox equivalent; the same letters in rows indicate the lack of a significant difference at *p* < 0.05.

**Table 2:** Phenolic compound profile and main groups of phenolics in red arils (mean ± SD).

Component	Fruit Collection Site			
	Zielona Góra	Warsaw	Koszalin	Cracow
Phenolic compounds determined by HPLC, µg/100 g of fresh weight				
*p*-Coumaric acid	80 ± 1 ^b^	70 ± 1 ^a^	130 ± 2 ^d^	100 ± 1 ^c^
Ferulic acid	40 ± 1 ^a^	130 ± 6 ^c^	100 ± 3 ^b^	120 ± 0 ^c^
Gallic acid	nd	100 ± 1 ^b^	120 ± 8 ^b,c^	140 ± 1 ^c^
Protocatechuic acid	nd	40 ± 1 ^c^	40 ± 2 ^c^	30 ± 0 ^b^
(-)-Epicatechin	1100 ± 20 ^a^	1070 ± 12 ^a^	1090 ± 15 ^a^	1050 ± 30 ^a^
Total polyphenols	1220 ± 10 ^a^	1410 ± 12 ^b^	1480 ± 14 ^c^	1440 ± 27 ^c^
Groups of phenolic compounds spectrophotometrically assayed, mg/100 g of fresh weight				
Flavonoids, mg C	8.5 ± 0.5 ^a^	21.1 ± 1.9 ^c^	12.2 ± 1.1 ^b^	11.0 ± 1.4 ^b^
Phenylpropanoids, mg CA	39.2 ± 3.3 ^a^	55.2 ± 6.6 ^b,c^	65.3 ± 5.5 ^c^	42.8 ± 1.7 ^a,b^
Flavonols, mg Q	28.8 ± 3.3 ^a^	45.5 ± 4.1 ^a,b^	53.6 ± 7.3 ^b^	34.1 ± 2.4 ^a^
Anthocyanins, mg Cy	33.3 ± 2.7 ^a^	59.3 ± 5.2 ^b^	39.0 ± 6.9 ^a^	35.5 ± 1.7 ^a^
Total polyphenols, mg GAE	25.7 ± 2.7 ^a^	53.8 ± 3.4 ^c^	49.8 ± 3.0 ^c^	34.4 ± 1.2 ^b^

nd—not detected; C—catechin; CA—caffeic acid; Q—quercetin; Cy—cyanidin; GAE—gallic acid equivalents; the same letters in rows indicate the lack of a significant difference at *p* < 0.05.

**Table 3:** Volatile compounds (%) of red arils (means ± SD).

Compound	Fruit Collection Site			
	Zielona Góra	Warsaw	Koszalin	Cracow
**Alcohols**				
Ethanol	8.93 ± 0.03 ^c^	7.36 ± 0.56 ^b^	6.27 ± 0.52 ^a^	8.55 ± 0.42 ^c^
1-Butanol	nd	nd	0.94 ± 0.04 ^b^	0.51 ± 0.03 ^a^
1-Pentanol	0.25 ± 0.00 ^a^	0.26 ± 0.01 ^a^	0.37 ± 0.01 ^c^	0.33 ± 0.01 ^b^
1-Hexanol	3.38 ± 0.06 ^a^	5.20 ± 0.68 ^b^	14.95 ± 0.41 ^d^	7.60 ± 0.38 ^c^
2-Hexanol	0.49 ± 0.03 ^b^	0.31 ± 0.01 ^a^	0.31 ± 0.02 ^a^	nd
1-Octanol	0.73 ± 0.02 ^a^	1.96 ± 0.28 ^c^	2.42 ± 0.08 ^d^	1.56 ± 0.06 ^b^
3-Octanol	0.10 ± 0.00 ^a^	0.43 ± 0.04 ^c^	0.31 ± 0.02 ^b^	0.59 ± 0.01 ^d^
1-Butanol, 3-methyl	3.73 ± 0.02 ^b^	1.91 ± 0.12 ^a^	3.84 ± 0.27 ^b^	1.92 ± 0.08 ^a^
1-Butanol, 2-methyl	2.90 ± 0.02 ^c^	1.51 ± 0.08 ^b^	2.87 ± 0.10 ^c^	1.28 ± 0.03 ^a^
3-Buten-1-ol, 3-methyl	0.33 ± 0.01 ^a^	0.33 ± 0.02 ^a^	0.39 ± 0.03 ^b^	nd
2-Buten-1-ol, 3-methyl	nd	0.37 ± 0.03 ^c^	0.34 ± 0.01 ^b^	0.31 ± 0.01 ^a^
2,3-Butanediol	16.35 ± 0.13 ^c^	0.59 ± 0.06 ^a^	2.12 ± 0.18 ^b^	nd
3-Hexen-1-ol	0.94 ± 0.16 ^a^	1.79 ± 0.23 ^b^	3.51 ± 0.09 ^c^	5.55 ± 0.36 ^d^
1-Octen-3-ol	0.14 ± 0.01 ^a^	0.64 ± 0.06 ^b^	0.63 ± 0.02 ^b^	0.72 ± 0.03 ^c^
1-Hexanol, 2-ethyl	0.17 ± 0.01 ^a^	nd	0.92 ± 0.02 ^b^	0.18 ± 0.01 ^a^
Benzenemethanol	2.10 ± 0.08 ^a^	3.64 ± 0.35 ^c^	5.17 ± 0.14 ^d^	2.91 ± 0.05 ^b^
Benzeneethanol	6.28 ± 0.12 ^d^	0.80 ± 0.09 ^a^	3.44 ± 0.17 ^c^	1.19 ± 0.03 ^b^
Total	46.80 ± 0.10 ^c^	27.09 ± 2.51 ^a^	48.77 ± 0.27 ^d^	33.20 ± 1.25 ^b^
**Ketones**				
3-Pentanone	nd	nd	1.02 ± 0.05	nd
3-Octanone	0.46 ± 0.02 ^a^	3.13 ± 0.41 ^c^	1.72 ± 0.16 ^b^	4.50 ± 0.27 ^d^
2,3-Butanedione	2.79 ± 0.02 ^b^	5.17 ± 0.35 ^c^	2.46 ± 0.11 ^b^	1.52 ± 0.03 ^a^
1-Octen-3-one	nd	0.26 ± 0.04 ^b^	nd	0.20 ± 0.01 ^a^
2-Butanone, 3-hydroxy	9.75 ± 0.08 ^d^	6.22 ± 0.51 ^c^	3.27 ± 0.24 ^b^	1.72 ± 0.04 ^a^
2,6,6-Trimethyl-2-cyclohexene-1,4-dione	3.37 ± 0.13 ^b^	6.13 ± 0.35 ^d^	2.37 ± 0.03 ^a^	4.72 ± 0.38 ^c^
Acetofenon	nd	0.11 ± 0.01 ^a^	nd	0.28 ± 0.02 ^b^
Total	16.37 ± 0.22 ^c^	21.02 ± 1.59 ^d^	10.83 ± 0.21 ^a^	12.93 ± 0.66 ^b^
**Esters**				
Ethyl acetate	5.58 ± 0.17 ^d^	2.14 ± 0.14 ^b^	4.95 ± 0.53 ^c^	0.78 ± 0.01 ^a^
Ethyl 2-methylbutanoate	0.65 ± 0.06 ^a^	nd	0.60 ± 0.03 ^a^	nd
Methyl hexanoate	0.09 ± 0.01 ^a^	0.11 ± 0.02 ^a^	0.25 ± 0.01 ^b^	0.35 ± 0.03 ^c^
Ethyl hexanoate	1.06 ± 0.02 ^b^	0.58 ± 0.08 ^a^	1.25 ± 0.07 ^b^	0.58 ± 0.21 ^a^
Hexyl acetate	0.11 ± 0.05 ^a^	nd	0.43 ± 0.02 ^b^	nd
Methyl octanoate	nd	nd	0.22 ± 0.01 ^b^	0.20 ± 0.01 ^a^
Ethyl benzoate	0.14 ± 0.05 ^a^	nd	0.21 ± 0.01 ^b^	nd
Ethyl octanoate	0.35 ± 0.12 ^a^	nd	0.33 ± 0.02 ^a^	nd
Methyl nonanoate	nd	nd	0.04 ± 0.00 ^a^	0.18 ± 0.02 ^b^
Ethyl nonanoate	0.11 ± 0.01 ^b^	nd	0.07 ± 0.01 ^a^	nd
Ethyl decanoate	0.05 ± 0.01	nd	nd	nd
Ethyl isobutanoate	0.19 ± 0.02	nd	nd	nd
Isopentyl acetate	0.38 ± 0.07 ^a^	nd	0.50 ± 0.02 ^b^	nd
Total	8.67 ± 0.31 ^c^	2.82 ± 0.20 ^b^	8.84 ± 0.71 ^c^	2.08 ± 0.24 ^a^
**Aldehydes**				
Hexanal	2.08 ± 0.05 ^c^	1.58 ± 0.11 ^a^	1.74 ± 0.12 ^a,b^	1.84 ± 0.04 ^b^
Heptanal	0.24 ± 0.03 ^a^	0.31 ± 0.01^b^	0.22 ± 0.01 ^a^	0.38 ± 0.01 ^c^
Nonanal	1.02 ± 0.08 ^b^	0.81 ± 0.08 ^a,b^	0.62 ± 0.07 ^a^	2.25 ± 0.20 ^c^
Decanal	0.27 ± 0.05 ^a,b^	0.33 ± 0.08^b^	0.18 ± 0.03 ^a^	0.23 ± 0.03 ^a^
2-Hexenal	nd	0.33 ± 0.03 ^a^	nd	0.37 ± 0.03 ^b^
3-Methylbutanal	nd	0.58 ± 0.04 ^b^	0.59 ± 0.03 ^b^	0.31 ± 0.02 ^a^
Benzaldehyde	1.10 ± 0.08 ^a^	3.42 ± 0.44 ^c^	2.37 ± 0.22 ^b^	1.85 ± 0.24 ^b^
Total	4.70 ± 0.14 ^a^	7.34 ± 0.77 ^c^	5.71 ± 0.23 ^b^	7.22 ± 0.36 ^c^
**Hydrocarbons**				
Toluene	nd	0.14 ± 0.03 ^a^	0.25 ± 0.02 ^b^	0.13 ± 0.01 ^a^
Styrene	nd	nd	0.59 ± 0.06	nd
Dodecane	0.14 ± 0.07 ^a^	0.39 ± 0.06 ^b^	0.31 ± 0.01 ^b^	0.34 ± 0.02 ^b^
2,4-Dimethylheptane	nd	nd	0.18 ± 0.01	nd
Total	0.14 ± 0.07 ^a^	0.53 ± 0.08 ^b^	1.33 ± 0.05 ^c^	0.47 ± 0.03 ^b^
**Terpenes and Terpenoids**				
α-Pinene	0.07 ± 0.02 ^a^	0.10 ± 0.00 ^a^	0.44 ± 0.02^c^	0.37 ± 0.04 ^b^
β-Pinene	0.23 ± 0.06 ^a^	0.46 ± 0.03 ^b^	0.47 ± 0.04 ^b^	0.22 ± 0.03 ^a^
m-Cymene	0.12 ± 0.03 ^a^	0.23 ± 0.03 ^b^	0.52 ± 0.05^c^	0.19 ± 0.02 ^b^
γ-Terpinene	0.06 ± 0.02 ^a^	0.13 ± 0.00 ^b^	0.48 ± 0.02^d^	0.32 ± 0.00^c^
3-Carene	nd	0.12 ± 0.03 ^a^	0.20 ± 0.04 ^b^	0.11 ± 0.01 ^a^
4-Carene	0.14 ± 0.06 ^a^	nd	0.39 ± 0.01 ^b^	0.23 ± 0.00 ^a^
D-Limonene	1.50 ± 0.48 ^a^	2.87 ± 0.23 ^b^	1.33 ± 0.05 ^a^	1.50 ± 0.28 ^a^
α-Terpinolen	nd	0.83 ± 0.08 ^b^	0.22 ± 0.02 ^a^	0.92 ± 0.01 ^c^
4-Carvomenthenol	nd	nd	0.33 ± 0.02	nd
4-Thujanol	nd	nd	0.58 ± 0.03	nd
Menthol	nd	nd	nd	0.34 ± 0.07
Myrtenol	nd	0.46 ± 0.04 ^a^	1.05 ± 0.08 ^b^	0.53 ± 0.02 ^a^
Total	2.07 ± 0.66 ^a^	5.18 ± 0.41 ^b^	6.00 ± 0.19^c^	4.65 ± 0.44 ^b^
**Lactones**				
Butyrolactone	0.45 ± 0.05 ^a^	0.80 0.28 ^b^	1.01 ± 0.07 ^b^	nd

nd—not detected; the same letters in rows indicate the lack of a significant difference at *p* < 0.05.

## Data Availability

Not applicable.

## Sample Availability

Samples of the compounds are not available from the authors.

## References

[1] Crespo P., Bordonaba J.G., Terry L.A., Carlen C. (2010). Characterisation of major taste and health-related compounds of four strawberry genotypes grown at different Swiss production sites. Food Chem..

[2] Ma X., Laaksonen O., Zheng J., Yang W., Trépanier M., Kallio H., Yang B. (2016). Flavonol glycosides in berries of two major subspecies of sea buckthorn (*Hippophaë rhamnoides* L.) and influence of growth sites. Food Chem..

[3] Demir N., Yildiz O., Alpaslan M., Hayaloglu A.A. (2014). Evaluation of volatiles, phenolic compounds and antioxidant activities of rose hip (*Rosa* L.) fruits in Turkey. LWT Food Sci. Technol..

[4] Vaneková Z., Vanek M., Škvarenina J., Nagy M. (2020). The influence of local habitat and microclimate on the levels of secondary metabolites in Slovak bilberry (*Vaccinium myrtillus* L.) fruits. Plants.

[5] Jaakkola M., Korpelainen V., Hoppula K., Virtanen V. (2012). Chemical composition of ripe fruits of *Rubus chamaemorus* L. grown in different habitats. J. Sci. Food Agric..

[6] Mannino G., Perrone A., Campobenedetto C., Schittone A., Margherita Bertea C., Gentile C. (2020). Phytochemical profile and antioxidant properties of *Plinia trunciflora* fruits: A new source of nutraceuticals. Food Chem..

[7] Lachowicz S., Seliga Ł., Pluta S. (2020). Distribution of phytochemicals and antioxidative potency in fruit peel, flesh, and seeds of Saskatoon berry. Food Chem..

[8] Mikulic-Petkovsek M., Schmitzer V., Slatnar A., Stampar F., Veberic R. (2015). A comparison of fruit quality parameters of wild bilberry (*Vaccinium myrtillus* L.) growing at different locations. J. Sci. Food Agric..

[9] Tabaszewska M., Rutkowska J., Skoczylas Ł., Słupski J., Antoniewska A., Smoleń S., Łukasiewicz M., Baranowski D., Duda I., Pietsch J. (2021). Red arils of *Taxus baccata* L.—A new source of valuable fatty acids and nutrients. Molecules.

[10] De Souza V.R., Pereira P.A., da Silva T.L., de Oliveira Lima L.C., Pio R., Queiroz F. (2014). Determination of the bioactive compounds, antioxidant activity and chemical composition of Brazilian blackberry, red raspberry, strawberry, blueberry and sweet cherry fruits. Food Chem..

[11] Siegle L., Pietsch J. (2018). Taxus ingredients in the red arils of *Taxus baccata* L. determined by HPLC-MS/MS. Phytochem. Anal..

[12] Gai Q.Y., Jiao J., Wang X., Liu J., Fu Y.J., Lu Y., Wang Z.Y., Xu X.J. (2020). Simultaneous determination of toxoids and flavonoids in twigs and leaves of three Taxus species by UHPLC-MS/MS. J. Pharmaceut. Biomed..

[13] Das B., Takhi M., Srinivas K.V.N.S., Yadav J.S. (1993). Phenolics from needles of himalayan *Taxus baccata*. Phytochemistry.

[14] Krauze-Baranowska M. (2004). Flavonoids from the genus *Taxus*. Z. Naturforsch. C. J. Biosci..

[15] Erdemoglu N., Sener B., Ozcan Y., Ide S. (2003). Structural and spectroscopic characteristics of two new dibenzylbutane type lignans from *Taxus baccata* L. J. Mol. Struct..

[16] Milutinović M.G., Stanković M.S., Cvetković D.M., Topuzović M.D., Mihailović V.B., Marković S.D. (2015). Antioxidant and anticancer properties of leaves and seed cones from European yew (*Taxus baccata* L.). Arch. Biol. Sci..

[17] Kucukboyaci N., Sener B. (2010). Biological activities of lignans from *Taxus baccata* L. growing in Turkey. J. Med. Plant Res..

[18] Lugasi A., Biró L., Hóvárie J., Sági K.V., Brandt S., Barna É. (2003). Lycopene content of foods and lycopene intake in two groups of the Hungarian population. Nutr. Res..

[19] Zhong L., Gustavsson K.E., Oredsson S., Głąb B., Yilmaz J.L., Olsson M.E. (2016). Determination of free and esterified carotenoid composition in rose hip fruit by HPLC-DAD-APCI^+^-MS. Food Chem..

[20] Szajdek A., Borowska E.J. (2008). Bioactive compounds and health-promoting properties of berry fruits: A review. Plant Foods Hum. Nutr..

[21] Cocco C., Magnani S., Maltoni M.L., Quacquarelli I., Cacchi M., Antunes L.E.C., D’Antuono L.F., Faedi W., Baruzzi G. (2015). Effects of site and genotype on strawberry fruits quality traits and bioactive compounds. J. Berry Res..

[22] Neelam, Khatkar A., Sharma K.K. (2019). Phenylpropanoids and its derivatives: Biological activities and its role in food, pharmaceutical and cosmetic industries. Crit. Rev. Food Sci. Nutr..

[23] Kilani-Jaziri S., Mokdad-Bzeouich I., Krifa M., Nasr N., Ghedira K., Chekir-Ghedira L. (2017). Immunomodulatory and cellular anti-oxidant activities of caffeic, ferulic, and p-coumaric phenolic acids: A structure-activity relationship study. Drug. Chem. Toxicol..

[24] Brzezinska E., Kozlowska M. (2008). Effect of sunlight on phenolic compounds accumulation in coniferous plants. Dendrobiology.

[25] Fukumoto L.R., Mazza G. (2000). Assessing antioxidant and prooxidant activities of phenolic compounds. J. Agric. Food Chem..

[26] Santos L.F.S., Stolfo A., Calloni C., Salvador M. (2017). Catechin and epicatechin reduce mitochondrial dysfunction and oxidative stress induced by amiodarone in human lung fibroblasts. J. Arrhythm..

[27] Tsanova-Savova S., Ribarova F., Gerova M. (2005). (+)-Catechin and (-)-epicatechin in Bulgarian fruits. J. Food Compos. Anal..

[28] Urpi-Sarda M., Monagas M., Khan N., Lamuela-Raventos R.M., Santos-Buelga C., Sacanella E., Castell M., Permanyer J., Andres-Lacueva C. (2009). Epicatechin, procyanidins, and phenolic microbial metabolites after cocoa intake in humans and rats. Anal. Bioanal. Chem..

[29] Drkenda P., Spahić A., Akagic A., Gasi F., Oras A., Hudina M., Blanke M. (2014). Pomological characteristics of some autochthonous genotypes of cornelian cherry (*Cornus mas* L.) in Bosnia and Herzegovina. Erwerbs-Obstbau.

[30] Chen G.-L., Chen S.-G., Zhao Y.-Y., Lou C.-X., Li J., Gao Y.-Q. (2014). Total phenolic contents of 33 fruits and their antioxidant capacities before and after *in vitro* digestion. Ind. Crops. Prod..

[31] Coklar H., Akbulut M. (2017). Anthocyanins and phenolic compounds of *Mahonia aquifolium* berries and their contributions to antioxidant activity. J. Funct. Foods..

[32] Popović Z.S., Matic R., Bajić-Ljubičić J., Tešević V., Bojovic S. (2018). Geographic variability of selected phenolic compounds in fresh berries of two *Cornus* species. Trees.

[33] Oszmiański J., Lachowicz S. (2016). Effect of the production of dried fruits and juice from chokeberry (*Aronia melanocarpa* L.) on the content and antioxidant activity of bioactive compounds. Molecules.

[34] Bialek M., Rutkowska J., Hallmann E. (2012). Black chokeberry (*Aronia melanocarpa*) as potential component of functional food. Żywn. Nauka Technol. Jakość..

[35] Zanfini A., Corbini G., La Rosa C., Dreassi E. (2010). Antioxidant activity of tomato lipophilic extracts and interactions between carotenoids and α-tocopherol in synthetic mixtures. LWT Food Sci. Technol..

[36] Kılıç Ö., Kocak A. (2014). Volatile constituents of *Juniperus communis* L., *Taxus canadensis* Marshall. and *Tsuga canadensis* (L.) Carr. from Canada. J. Agric. Sci. Technol..

[37] Stefanović M., Ristić M., Popović Z., Matić R., Nikolić B., Vidaković V., Obratov-Petković D., Bojković S. (2016). Chemical composition and interpopulation variability of essential oils of *Taxus baccata* L. from Serbia. Chem. Biodivers..

[38] Choi J.Y., Lee S.M., Lee H.J., Kim Y.-S. (2018). Characterization of aroma-active compounds in Chinese quince (*Pseudocydonia sinensis* Schneid) by aroma dilution analyses. Food Res. Int..

[39] El Hadi M.A., Zhang F.J., Wu F.F., Zhou C.H., Tao J. (2013). Advances in fruit aroma volatile research. Molecules.

[40] Ye L., Yang C., Li W., Hao J., Sun M., Zhang J., Zhang Z. (2017). Evaluation of volatile compounds from Chinese dwarf cherry (*Cerasus humilis* (Bge.) Sok.) germplasms by headspace solid-phase microextraction and gas chromatography-mass spectrometry. Food Chem..

[41] Vitova E., Divišová R., Sůkalová K., Matějíček A. (2013). Determination and quantification of volatile compounds in fruits of selected elderberry cultivars grown in Czech Republic. J. Food Nutr. Res..

[42] Serradilla M.J., Martín A., Ruiz-Moyano S., Hernández A., López-Corrales M., de Guía-Córdoba M. (2012). Physicochemical and sensorial characterisation of four sweet cherry cultivars grown in Jerte Valley (Spain). Food Chem..

[43] Amanpour A., Sonmezdag A.S., Kelebek H., Selli S. (2015). GC-MS-olfactometric characterization of the most aroma-active components in a representative aromatic extract from Iranian saffron (*Crocus sativus* L.). Food Chem..

[44] Maggi L., Carmona M., Zalacain A., Kanakis C.D., Anastasaki E., Tarantilis P.A., Polissiou M.G., Alonso G.L. (2010). Changes in saffron volatile profile according to its storage time. Food Res. Int..

[45] Vendramini A.L., Trugo L.C. (2000). Chemical composition of acerola fruit (*Malpighia punicifolia* L.) at three stages of maturity. Food Chem..

[46] Sampaio T.S., Nogueira P.C.L. (2006). Volatile components of mangaba fruit (*Hancornia speciosa* Gomes) at three stages of maturity. Food Chem..

[47] Oliveira I., Guedes de Pinho P., Malheiro R., Baptista P., José A., Pereira J.A. (2011). Volatile profile of *Arbutus unedo* L. fruits through ripening stage. Food Chem..

[48] Rozza A.L., Moraes T.d.M., Kushima H., Tanimoto A., Marques M.O., Bauab T.M., Hiruma-Lima C.A., Pellizzon C.H. (2011). Gastroprotective mechanisms of Citrus lemon (*Rutaceae*) essential oil and its majority compounds limonene and β-pinene: Involvement of heat-shock protein-70, vasoactive intestinal peptide, glutathione, sulfhydryl compounds, nitric oxide and prostaglandin E_2_. Chem. Biol. Interact..

[49] Kuttan G., Pratheeshkumar P., Manu K.A., Kuttan R. (2011). Inhibition of tumor progression by naturally occurring terpenoids. Pharm. Biol..

[50] Gutiérrez-Del-Río I., Fernández J., Lombó F. (2018). Plant nutraceuticals as antimicrobial agents in food preservation: Terpenoids, polyphenols and thiols. Int. J. Antimicrob. Agents..

[51] Sepici-Dincel A., Açikgöz S., Cevik C., Sengelen M., Yeşilada E. (2007). Effects of *in vivo* antioxidant enzyme activities of myrtle oil in normoglycaemic and alloxan diabetic rabbits. J. Ethnopharmacol..

[52] Badary O.A. (1999). Thymoquinone attenuates ifosfamide-induced Fanconi syndrome in rats and enhances its antitumor activity in mice. J. Ethnopharmacol..

[53] Singleton V.L., Rossi J.A. (1965). Colorimetry of total phenolics with phosphomolybdic- phosphotungstic acid reagents. Am. J. Enol. Vitic..

[54] Ardestani A., Yazdanparast R. (2007). Antioxidant and free radical scavenging potential of *Achillea santolina* extracts. Food Chem..

[55] Zhishen J., Mengcheng T., Jianming W. (1999). The determination of flavonoid contents in mulberry and their scavenging effects on superoxide radicals. Food Chem..

[56] Polish Standard (2000). Fruit and Vegetable Juice-Total Carotenoids and Carotenoids Fraction Determination (PN-EN 12136).

[57] Nagata M., Yamashita I. (1992). Simple method for simultaneous determination of chlorophyll and carotenoids in tomato fruit. Nippon Shokuhin Kogyo Gakkaish.

[58] European Standard (2003). Foodstuffs—Determination of Vitamin C by HPLC (PN-EN 14130).

[59] Klimczak I., Małecka M., Szlachta M., Gliszczyńska-Świgło A. (2007). Effect of storage on the content of polyphenols, vitamin C and the antioxidant activity of orange juices. J. Food Compos. Anal..

[60] Skoczylas Ł., Tabaszewska M., Smoleń S., Słupski J., Liszka-Skoczylas M., Barański R. (2020). Carrots (*Daucus carota* L.) biofortified with iodine and selenium as a raw material for the production of juice with additional nutritional functions. Agronomy.

[61] Sánchez-Moreno C., Larrauri J.A., Saura-Calixto F.A. (1998). Procedure to measure the antiradical efficiency of polyphenols. J. Sci. Food Agric..

[62] Re R., Pellegrini N., Proteggente A., Pannala A., Yang M., Rice-Evans C. (1999). Antioxidant activity applying an improved ABTS radical cation decolorization assay. Free Radic. Biol. Med..

[63] Serpen A., Gökmen V., Pellegrini N., Fogliano V. (2008). Direct measurement of the total antioxidant capacity of cereal products. J. Cereal Sci..

[64] Benzie I.F., Strain J.J. (1996). The ferric reducing ability of plasma (FRAP) as a measure of “antioxidant power”: The FRAP assay. Anal. Biochem..

